# The Role of 8-Amidoquinoline Derivatives as Fluorescent Probes for Zinc Ion Determination

**DOI:** 10.3390/s21010311

**Published:** 2021-01-05

**Authors:** Nur Syamimi Mohamad, Nur Hanis Zakaria, Nurulhaidah Daud, Ling Ling Tan, Goh Choo Ta, Lee Yook Heng, Nurul Izzaty Hassan

**Affiliations:** 1 Southeast Asia Disaster Prevention Research Initiative (SEADPRI-UKM), Institute for Environment and Development (LESTARI), Universiti Kebangsaan Malaysia, Bangi 43600, Selangor, Malaysia; p90391@siswa.ukm.edu.my (N.S.M.); lingling@ukm.edu.my (L.L.T.); gohchoota@ukm.edu.my (G.C.T.); 2 Department of Chemical Sciences, Faculty of Science & Technology, Universiti Kebangsaan Malaysia, Bangi 43600, Selangor, Malaysia; p97344@siswa.ukm.edu.my (N.H.Z.); yhl1000@ukm.edu.my (L.Y.H.); 3 Pusat GENIUS@Pintar Negara, Universiti Kebangsaan Malaysia, Bangi 43600, Selangor, Malaysia; nurulhaidah@ukm.edu.my

**Keywords:** zinc sensor, 8-amidoquinoline, fluorescent probe, chemosensor, systematic review

## Abstract

Mass-spectrometry-based and X-ray fluorescence-based techniques have allowed the study of the distribution of Zn^2+^ ions at extracellular and intracellular levels over the past few years. However, there are some issues during purification steps, sample preparation, suitability for quantification, and the instruments’ availability. Therefore, work on fluorescent sensors based on 8-aminoquinoline as tools to detect Zn^2+^ ions in environmental and biological applications has been popular. Introducing various carboxamide groups into an 8-aminoquinoline molecule to create 8-amidoquinoline derivatives to improve water solubility and cell membrane permeability is also a recent trend. This review aims to present a general overview of the fluorophore 8-aminoquinoline and its derivatives as Zn^2+^ receptors for zinc sensor probes. Various fluorescent chemosensor designs based on 8-amidoquinoline and their effectiveness and potential as a recognition probe for zinc analysis were discussed. Based on this review, it can be concluded that derivatives of 8-amidoquinoline have vast potential as functional receptors for zinc ions primarily because of their fast reactivity, good selectivity, and bio-compatibility, especially for biological applications. To better understand the Zn^2+^ ion fluorophores’ function, diversity of the coordination complex and geometries need further studies. This review provides information in elucidating, designing, and exploring new 8-amidoquinoline derivatives for future studies for the improvement of chemosensors that are selective and sensitive to Zn^2+^.

## 1. Introduction

Zinc ion plays a significant role in biological and pathological processes [1,2,3]. The past decade has seen many excellent metal-ion sensors detect transition and heavy metal ions such as zinc ions Zn^2+^. Zn^2+^, as an essential element in the human body, is actively involved in various biochemical processes such as neuronal signal transmission, DNA binding, enzyme regulators, and catalytic centers. Zn^2+^ deficiency and imbalance distribution then lead towards a much broad range of pathologies in parts of body systems [4,5]. The deficiency of Zn^2+^ in the human body can lead to severe neurological disorders like Alzheimer’s. In contrast, an excess of Zn^2+^ reported as an effect of excessive consumption in time can lead to Parkinson’s disease [6,7,8]. According to the US Food and Nutritional Board, the human body can receive a safe amount of Zn^2+^ in a range of 2 mg (0–5 years) to 15 mg (adults) [9].

## 2. Significance, Development, and Challenges in the Detection of Zinc Ions with Various Analytical Methods

In the past several years, mass-spectrometry-based, and X-ray fluorescence-based techniques facilitating the analysis of Zn^2+^ ions distribution at extracellular and intracellular levels. Measuring this trace element’s composition in those cells was crucial in understanding the role of Zn^2+^ ions in biological and pathological, as mentioned above. The researchers could also measure free Zn^2+^ ions, whether in deficiency or excess. Even though the body system precisely needs Zn^2+^ ions to operate very well, the excess amount of Zn^2+^ could lead to toxicity to body functions. Some challenges in monitoring free zinc at and below nanomolar levels are widely appreciated, such as potential interference at higher concentrations with Ca^2+^ and Mg^2+^.

Among the analytical mass-spectrometry methods that have been widely applied in research of measuring zinc in biological samples are inductively coupled plasma mass spectrometry (ICP-MS) and secondary-ion mass spectrometry (SIMS) [10]. Both instruments are powerful analytical technologies for elemental and isotopic analysis. The method comprises dissolving the organs, purifying the chemical zinc from the rest of the atoms, and then measuring the isotope ratio to instruments. With multi collector-ICP-MS (MC-ICP-MS), zinc isotope presence is determined in various organ samples of rats to comprehend zinc metabolism on the subject of a potential biomarker for Alzheimer’s disease [11]. In another study, they use laser ablation-MC-ICP-MS (LA-MC-ICP-MS) to measure Zn^2+^ isotope incorporation in rat brain thin sections to identify alteration zinc’s homeostasis that has been connected to neuronal signal transmission [12,13]. You and co-workers also studied portions of brain tissue (hypothalamus, hippocampus, cortex, and amygdala). They found that the ratio of ^31^P/^66^Zn decreased beyond tumor limits, indicating that variations in this ratio can be used to distinguish healthy vs. cancerous tissue [14].

Weiskirchen and co-workers did further examples examining metal’s concentration as disease biomarkers using LA-ICP-MS in identifying diseased liver status (diseased liver tissue: Levels of Fe and Cu higher while levels of Zn^2+^ levels lower) [15]. So, if the concentration of Zn^2+^ ions in that liver tissue suddenly drops from the supposed amount of Zn^2+^ ions in normal tissue, liver disease is detected. In other words, this condition could indicate the turn from ordinary to unhealthy tissue. However, as mentioned above, chemical zinc in the target organs needed to be purified first before analyzing with the mass spectrometry. At this stage, they are some difficulties and issues during the purification step. The percentage of purity may lead to false results.

Secondary ion-MS (SIMS) is another analytical mass-spectrometry method used for imaging trace elements and their isotopes. As for high resolution-SIMS (NanoSIMS), it offers excellent sensing at 50 nm to a few microns [16]. The localization of Zn and Cd in Poplar species (Migeon and co-workers) and radish (Ondrasek and co-workers) grown in metal contaminated soil were analyzed by NanoSIMS. Both studies showed that the micronutrient Zn and the toxic Cd were readily transported to the plants from the polluted soils [16,17]. Both metals have already translocated from the rhizosphere to edible hypocotyl radish and shoot tissues at a comparable pace, also under short-term exposure [16]. These research types help comprehend plants’ physiology and imply irrigation and biomonitoring of water and soil pollution. However, NanoSIMS is not suitable as a quantification tool. Nevertheless, the preparation of the samples is quite challenging. The samples need to be in a vacuum. 

Synchrotron X-ray fluorescence (SXRF) is usually non-destructive and needs no vacuum instead of mass spectrometry-based techniques. Recently, McRae and co-workers monitored the location and redistribution of phosphorus (P), sulfur (S), iron (Fe), copper (Cu), and zinc (Zn) during mitosis of mammalian cells by using SXRF fluorescence. The colocalization analysis distribution of Zn-S levels increased in mitosis compared to the interphase stage. They suggested that this increased zinc amount showed zinc’s vital role in the cell cycle [18]. This powerful method offered submicron spatial resolution and high sensitivity, but access to this instrument is limited due to the price. 

Mammalian egg fertilization initiates a series of ‘zinc sparks’ needed to induce the egg-to-embryo. Some studies used a combined approach to understand the importance of zinc-efflux in the molecular mechanisms by examining zinc distributions in a single cell at a different stage (maturation of egg: prophase I, breakdown of germinal vesical, metaphase I, anaphase I, telophase I dan metaphase II) [19,20]. As we mentioned above, zinc plays a vital role in biological systems, whether in plants or humans. However, the process(es) of the distribution pathway of the metals remains unexplored. It is necessary to determine free zinc concentration in cells for some reasons: (1) level of exchangeable Zn will express the zinc activity in the system; (2) free zinc levels help identify thresholds of high and low zinc toxicity according to theoretical concentrations in intracellular and extracellular at different stages; (3) free zinc concentrations can provide an overall measure of zinc nutrition, which is eventually useful for deficiency assessment; (4) understanding the roles of zinc in pathological statuses and courses, such as seizures, oxidative stress, amyloid plaque formation, ischemia, and apoptosis [21]. However, these zinc detections usually interfere with the other ions present in the system, such as Mg^2+^, Ca^2+^, Cu^2+^, and Fe^2+/3+^.

## 3. Zn^2+^ Fluorophore Based Sensors for the Analysis of Zn Ions

Chemical sensors, or chemosensors, are devices used for sensing target compounds by converting chemical information into “analytical” information. These analytical terms will imply the notion of measurability, whether in the qualitative or quantitative form [3]. A chemosensor has two essential parts as a receptor. It binds the substrate and a transducer that expresses the associated binding event [1,2,9], thus allowing a selective detection of numerous metal ions in various environmental and biological samples. Molecular recognition of chemosensor involves incorporating a chromophore or fluorophore as a binding site for analyte and consists of a mechanism of binding interactions between them [22,23]. Therefore, chemosensors play an essential role in detecting various analytes in many diverse fields, especially in analytical chemistry, bio- medicinal science, and environmental chemistry [24,25,26,27,28,29]. Multiple types of chemosensors, including fluorescent-type sensors, use spectrophotometry and fluorimetry to detect analytes [30,31,32,33,34,35,36,37,38,39]. 

Development of simple design and easy to synthesize fluorescent sensors with high selectivity recognizing metal ion is widely studied for its promising advantages. As ion-induced sensors, a fluorescent probe is highly useful for various applications such as in vivo and in vitro analyses of biologically essential species, including metal ions. It offers many advantages over other techniques because of its high sensitivity, repeatability, selectivity, and instantaneous response, offering less detection [31,32,33,34,35,36,39]. Despite the progress of fluorescent chemosensors in various detection, several problems and challenges continue to exist, leading to an exploration of the development of binding receptors. 

Some severe facts should be aware of this fluorescence sensor. First, the result’s observation is between the fluorescent sensor and target or the outcome affected by other factors like microenvironment, states, and temperature, especially on the first application. Second, the fluorescence probe should be distributed in uniform, but it is hard to be implemented experimentally. Third, the degradation of the fluorescent probe would lead to misunderstanding of the result. Fourth, the behavior of the sensor in solubility and cell permeability. Lastly, the need to be explored since this fluorescent chemosensor is analytical measuring in detecting not explaining unless combined with the other instrument.

A fluorescent chemosensor signal is typically calculated as changes of fluorescence intensity and/or transition of fluorescence wavelength, especially before and after introducing the probe to the target [40]. Until now, this fluorescence activity that involves cations and anions has been enabled by several mechanisms, such as internal charge transfer (ICT), chelation enhanced fluorescence (CHEF), photoinduced electron transfer (PET), and deprotonation mechanism [41]. By these mechanisms, the chemosensor’s fluorescence intensity could be quenched or enhanced depends on the probe. Several compounds had been studied along with the mechanism involved to get a better understanding of fluorescent chemosensors. Table 1 shows the comparisons and assessments of collected studies that related to the fluorescence probe.

Quinoline is an exciting compound with low initial fluorescence but can form highly fluorescent complexes with metal ions chosen as a fluorophore [42]. Quinoline is a unique fluorophore due to a relatively smaller molecular size, and the nitrogen in the ring makes it formed good metal coordination. Moreover, the quinoline ring itself also provides coordination quinoline-based metal-ligand binding by π-π intermolecular stacking ability. Analytically, this quinoline receptor is not limited to detecting Zn^2+^ only, and it also exhibits excellent coordination with the other metal cation like Cd^2+^, Hg^2+^, Al^3+^, Cr^3+^, Cu^2+^, Fe^3+^, and of course Zn^2+^ [43]. However, most of the quinoline-based derivatives were selective to Zn^2+^ ion and slightly interfered with the other metal ion. Therefore, the development of the quinoline fluorophore database is ongoing.

**Table 1:** The comparisons and assessments of collected studies that related to the fluorescence probe.

Article/Year	Analyte	Binding Mode	Sensing Mechanism	Fluorescence Signals	Detection Limits
[44]/2015	Zn^2+^	Lower rim amide linked 8-amino quinoline acts as a receptor molecule, and 8-amino naphthalene moiety 1,3,5-triderivatives of calix[6]arene acts as a control molecule	Absorption and Electrospray Ionization (ESI) MS Spectra	390 and 490 nm	-
[45]/2016	CEA	Carcinoembryonic antigen aptamer (5′-nh2-ataccagcttattcaatt-3′) conjugated to hexanedioic acid (hda) modified ucps (hda-ucps) by edc-nhs coupling method.	Fluorescence Resonance Energy Transfer (FRET)	-	0.8 pg/mL
[46]/2015	Zn^2+^ and Cu^2+^	Asymmetrical Diarylethene As Photoswitchable Core and Amidoquinoline As A Multi-Responsive Group Via A Piperazine Linkage	Fluorescence Chemosensor	417 nm to 502 nm	-
[47]/2018	Zn^2+^	N’-(quinolin-8-ylmethylene)benzohydrazide group as the binding unit and perfluorodiarylethene as a photoswitching trigger	Fluorescence Sensor	412 nm	3.2 × 10^−8^ mol L^−1^
[48]/2011	Zn^2+^ and Cu^2+^	Amide tautomerization	Fluorescence Sensor	492 to 430 nm	0.14 and 0.86 μm
[49]/2014	Zn^2+^	4-amino-1,8-naphthalimide-pet, with iminodiacetic acid as a chelating metal group	PET-Fluorescent Sensor	470 nm	-
[50]/2013	Zn^2+^ and Cu^2+^	2-((benzylimino)- methyl)-naphthalen-1-ol	PET-Fluorescence Sensor	300 nm, 370 nm	0.35 and 0.82 µm
[51]/2017	Al^3+^	Coumarin-Derived Chemosensor with 2-Hydroxy-4-Methylbenzohydrazide and Acetylcoumarin	Fluorescence Chemosensor	490 nm	6.7 μm

Derivatives of 8-hydroxyquinoline and 8-aminoquinoline are common fluorogenic chelators for Zn^2+^ ions [40]. Mummidivarapu and co-workers synthesized 8-amino quinoline and 8-amino naphthalene moiety 1,3,5-triderivatives of calix[6]arene and studied their binding ability toward biologically essential metal ions. They observed a stronger binding of Zn^2+^ from the absorption study, along with the complexation energies computed from the computational data [44]. A susceptible sensor was also reported by using amidoquinoline as part of the recognition element towards glucosamine in living Caco-2 cells, resulted in the enhancement of fluorescence quantum yields by 14 folds [45]. 

Another highly selective probe by Xia and co-workers used the diarylethene compound with a piperazine-linked amidoquinoline unit as a binder for zinc ions [46]. They reported a higher binding affinity of the complex with the targeted analyte in fluorescence intensity. These showed the derivatives of 8-aminoquinoline in the presence of amide gradually to be acknowledged. Several studies focusing on improving and increasing the sensitivity and selectivity of fluorescent probes for Zn^2+^ based on 8-amidoquinoline and its derivatives are widely developed [47,48]. Further, 8-amidoquinoline derivatives exhibit fluorescence properties for sensing Zn^2+^ based on important signaling mechanisms and internal charge transfer (ICT) [48,52]. Therefore, this review aims to assess collected studies related to 8-amidoquinoline derivatives as fluorophores of zinc chemosensor.

## 4. Quinoline and Derivatives for Zn^2+^ Fluorophores

The global focus has recently been devoted to developing and synthesizing highly sensitive fluorescent probes for the selective recognition of Zn^2+^ ions as a concern for human health and environmental safety. In total, 41 titles and abstracts were screened independently based on the criteria “practices of 8-aminoquinoline derivatives in the detection of zinc only”. Next, the left articles were evaluated for inclusion or exclusion based on 8-aminoquinoline with amide structure, or we might be called it 8-amidoquinoline or N-(quinolin-8-yl) formamide. The addition of the carboxamide group, followed by intramolecular hydrogen bond breaking of 8-aminoquinoline, inhibits intramolecular electron-transfer, which in turn enhanced the ICT process as pronounced in the Zhang [53] and Xu [54] study. This observation would enhance fluorescence emission.

**Figure 1:**
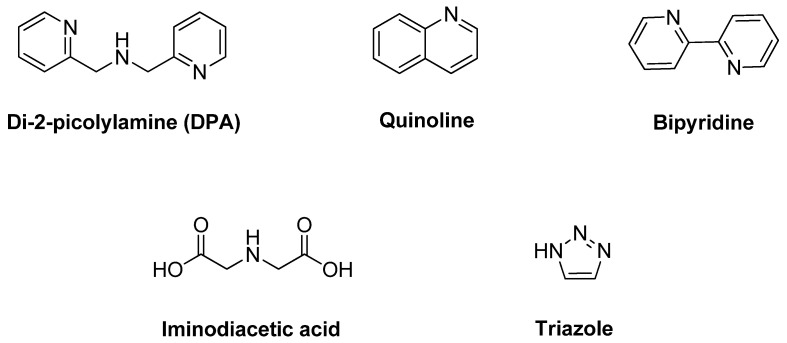
The frequent main structure of fluorophore probe used for binding site or receptors in fluorescence chemosensor. (as di-2-picolylamine (DPA, quinoline, bipyridine, iminodiacetic acid, and triazole).

The devotion to the spectroscopically silent behavior of the Zn^2+^ ion leads to interferences from the other metal ions. Aside from these interferences, the sensing results were influenced by the other factors such as the environment of the system including solvent used, type and pH of the buffer, the concentration of Zn^2+^ ions present compared to the other ions, solubility properties, and of course, the characteristic and behavior of the probe itself. They are so many receptors used as fluorophore as binding sites such as di-2-picolylamine (DPA) [55,56] quinoline [57,58], bipyridine [59,60,61,62], acyclic and cyclic polyamines [63,64], iminodiacetic acid [65,66], triazole [67,68,69], and Schiff-base receptors [70] (Figure 1). 

**Figure 2:**
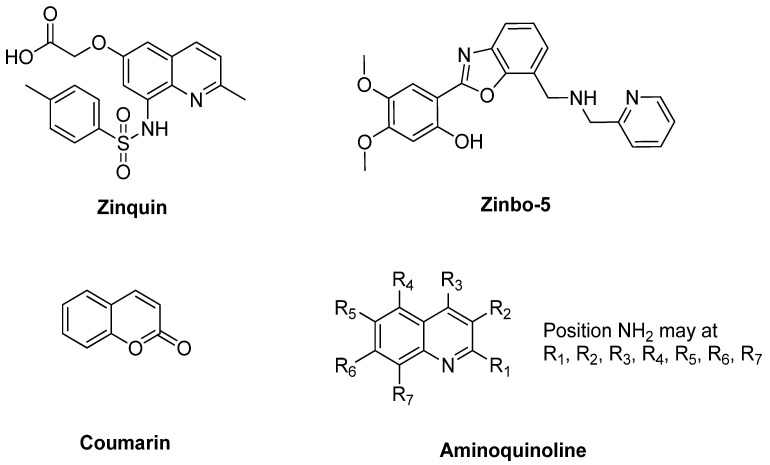
Structure of the earliest probe for zinc detection (Zinquin, Zinbo-5, coumarin and aminoquinoline 8-amidoquinoline).

However, it is necessary to notice that few compounds fluoresce by itself or fluoresce after the interaction with the target. The behavior of the compounds usually can electronically go to an excited and ground state during inter-intramolecular transfer. Thus, the most intense and valuable fluorescence is generally seen in compounds that comprise aromatic functional groups since they have a π system orbital [71]. Therefore, the earliest probes for zinc (II) detection are derivatives of zinquin [72], Zinbo-5 [73], Zinpyr [74], aminoquinoline [75], and coumarin families [76] (Figure 2).

### Aminoquinoline as a Fluorophore for Zinc’s Recognition

**Figure 3:**
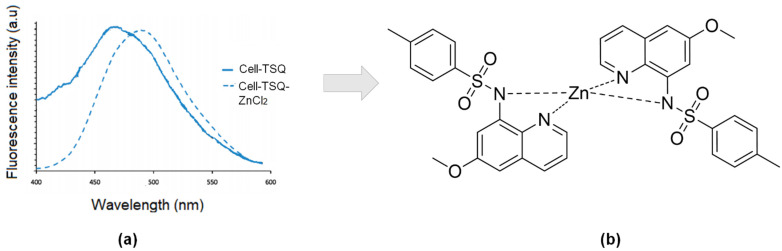
(**a**) Relative fluorescence of cells stained 30 min with 30 μMTSQ (blue), then following addition of 30 μM ZnCl_2_ (dotted line). (**b**) Possible complexation of 6-methoxy-(8-p-toluenesulfonamido)quinoline (TSQ)_2_-Zn.

An aryl sulfonamide derivative of 8-aminoquinoline, 6-methoxy-(8-p-toluenesulfonam -ido)quinoline (TSQ) is amongst the most efficient used as fluorophore in zinc sensor, especially in the biological field [21]. This TSQ was first published in the early seventies by Toroptsey and Eshchenko [77]. Then, referring to Meeusen and co-workers, TSQ acts as a chelating ligand to Zn^2+^ by a ratio of 2 to 1 ((TSQ)_2_Zn), which will form a metal complex. TSQ-Zn will increase the fluorescence intensity of TSQ by 4-fold (excitation maximum: 360 nm, emission maximum: 490 nm). They also indicate a blue-shifted emission spectrum as a ternary TSQ-Zn-protein adduct (Figure 3) [78]. Moreover, this TSQ has been used as a stain to recognized Zn^2+^ ions for biological purposes since it still provides selectivity over concentrations of Ca^2+^ and Mg^2+^ ions [43,79].

TSQ derivatives are extensively used as fluorogenic chelators in the detection of zinc in biological samples. However, these derivatives have low water solubility, poor membrane penetrability, and complicating free zinc ion measuring in cells quantitatively [41]. Therefore, to overcome those problems, developing on the quinoline-based derivatives still ongoing until now. This is due to their behavior high soluble in water and showed excellent selectivity for Zn^2+^ ion [80] as we can say that the importance of zinc detection almost in biological and environmental applications. Therefore, among other fluorophores, quinoline-based derivatives are studied extensively. 

**Figure 4:**
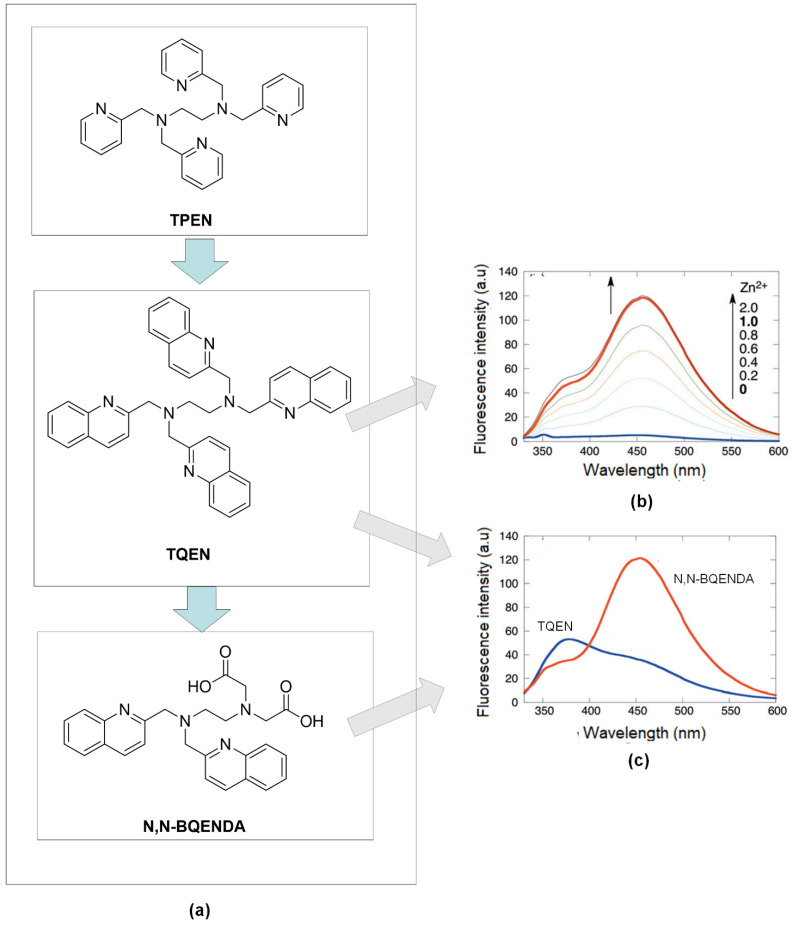
(**a**) Development zinc’s fluorophore from N,N,N’,N’-tetrakis(2-pyridylmethyl)ethylenediamine (TPEN) to N,N,N’,N’-tetrakis(2-quinolylmethyl) ethylenediamine (TQEN) and N,N-bis(2-quinolylmethyl) ethylenediamine- N’,N’-diacetic acid (N,N-BQENDA). (**b**) Fluorescence emission of TQEN upon increasing amount of Zn^2+^. (**c**) Comparison fluorescence emission of TQEN-Zn and N,N-BQENDA-Zn at ration 1:1 (DMF-Water).

As confirmation, the characteristic of quinoline on the zinc detection, Mikata, and co-workers substituted pyridine ring with quinoline group in N, N, N, N-tetrakis(2-pyridylmethyl) -ethylenediamine, (TPEN) to form N, N, N, N-tetrakis(2-quinolylmethyl) ethylenediamine (TQEN) compound (Figure 4a). Then, they modified the combination with the addition of the methoxy group. Those compounds are selective to Zn^2+^ by photoinduced electron-transfer (PET) and chelation enhanced fluorescence (CHEF). The modified combination of TQEN showed strong fluorescence upon binding with Zn^2+^ compared to the weak fluorescence of TPEN [81]. Steric and stereochemical effects are the key factors of metal-binding affinity. The aromatic ring of quinoline can be versatile and exhibit different affinities to the other metal-ligand binding. In the development of Zn^2+^ chelators, N-containing ligands like quinoline have been developed and investigated [82]. 

It is crucial to keep in mind that fluorescent groups with lone pairs of electrons like nitrogen will be quenching the emission. However, the coordinate bond formed between that lone pairs of electrons and metal ions like Zn^2+^ ion can prevent fluorescence from quenching. Hence, those nitrogen-containing heterocyclic chromophores whose nitrogen will form a chelate ring with metal ions will give a CHEF effect [71]. Upon excitation, the electron donor from the chelator to the acceptor will permit intramolecular charge transfer (ICT), preceded by a large Stokes’ shift [41]. This chemosensor design would be valued as a wavelength-shifted fluorescence intensity enhancement that will amplify the recognition event. 

Compared to quinoline isomer TQEN, isoquinoline derivates of TQEN exhibited strong fluorescence upon binding with Zn^2+^, and the isoquinoline (benzene ring position) showed higher metal binding affinity [83]. Next, hybrids of (N, N-bis(2-quinolylmethyl)ethylenediamine-N’, N’-diacetic acid (N, N-BQENDA) (Figure 4b), shows a 32-fold emission enhancement at 456 nm in buffer 4-(2-Hydroxyethyl)piperazine-1-ethane sulfonic acid, HEPES (pH = 7.5). The substitution of carboxylate groups into two quinolines in TQEN reduced the steric hindrance in forming metal ligands binding between Zn^2+^ and remaining N-atoms of quinolines. Figure 4c showed, under the same condition, the emission of N, N-BQENDA-Zn is more enhanced than the emission of TQEN-Zn. With the stronger metal bonding affinity, the sensor has higher selectivity towards Zn^2+^ detection [84].

**Figure 5:**
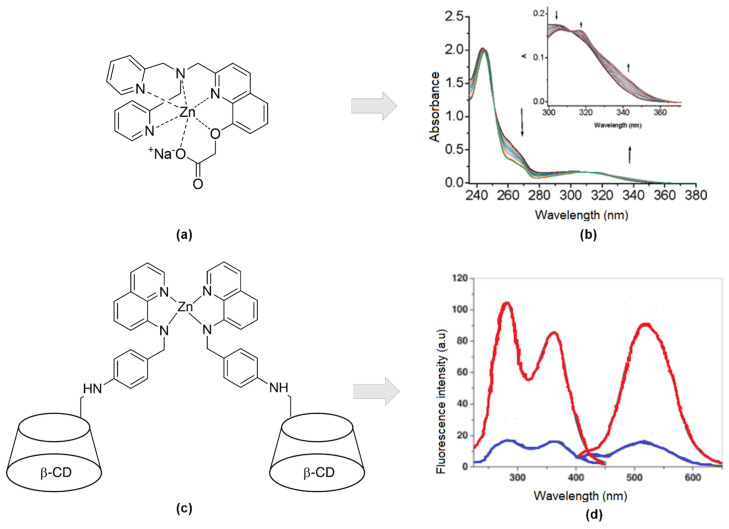
(**a**) Hexadentate of sodium 2-((2-((bis(pyridin-2-ylmethyl)amino)methyl)quinolin-8-yl)oxy)acetate (1) ligand-Zn; (**b**) Absorbance upon binding 0–1 equivalent to Zn^2+^ (25 mM HEPES, 0.1 M NaClO_4_, pH 7.4, 25 °C). Inset: Focused absorbance between 300 to 370 nm. (**c**) Covalently linked 6-deoxy-6-formyl-ß-cyclodextrin(CD) into an analog of TSQ, N-(8-quinolyl)-p-aminobenzene -sulfonamide (HQAS)-Zn. (**d**) Excitation and emission without Zn^2+^ (blue), with Zn^2+^ (red) at λ_ex_ = 285 nm and 362 nm, λ_em_ = 507 nm.

Another study using quinolines scaffold as zinc’s receptor was reported by Wang and co-workers [85]. They develop a new small molecular organic compound with excellent bonding affinity and high selectivity towards Zn^2+^ ions compared to other divalent metals. They combined 8-hydroxy-2-methyl quinoline (Oxn) that had a building block for the CHEF process to take place once binding with metals with potent chelators like di-2-picolylamine (DPA) moiety at the 2-position (1) (Figure 5a). Then, further saponification is conducted to get a carboxylic moiety for increasing the solubility of the compound in water. UV-Vis, fluorescent, and NMR titrations indicated tight binding between the compound and Zn^2+^ ion. Figure 5b showed three isosbestic’s points at 245, 250, and 312 nm, suggesting forming only one UV active zinc complex. Moreover, X-ray diffraction revealed zinc coordinated with quinoline, carboxylic moiety, and two pyridyl moieties. Bond lengths of Zn-N are reported, like the bond lengths of other Zn-DPA complexes [86,87,88].

Ligand 8-hydroxyquinoline (8-HQ), an established chromophore for zinc chelation, became the starting material as three tripodal ligands for zinc detection in research studied by Royzen and co-workers. All these three ligands were reported bound with Zn at a ratio of one to one. Strong fluorescent emission for all ligands (λmax 1 = 526 nm, λmax 2 = 513 nm, λmax 3 = 486 nm). Moreover, enhancement for ligands 2 and 3 showed 13.1 folds and 11.3 folds, respectively. X-ray crystallographic of these ligands indicated different molecular geometries of Zn-complex formation might influence their spectroscopic interactions of metal-ligands [89]. 

As TSQ had low solubility in water, several attempts had been made to overcome those properties, including diverting to quinoline-based derivatives, introducing carboxylic acid or ester to the extent of replacing methyl in benzene with a carboxylate group, and adding hydroxyl group as mentioned above. Other research groups take the initiative to covalently linked 6-deoxy-6-formyl-ß-cyclodextrin (CD) into an analog of TSQ, N-(8-quinolyl)-p-aminobenzene -sulfonamide (HQAS) (Figure 5c). As we can see, Figure 5d, there was two bands wavelength for excitation (285 nm and 362 nm) while the emission was emitted at 507 nm. For both situations (with/out the existence of Zn), the peak appeared at the same wavelength. The only difference just the intensities. The fluorescence intensity is enhanced because the electron-process was forbidden as per binding, and the π-electron conjugation system was involved in ICT from donor to acceptor [90]. This process will lead from weak fluorescence to strong fluorescence. They reported slight interferences of several metal ions ordinarily present in a physiological environment, such as Na^+^, K^+^, Ca^2+^, Mg^2+^, Mn^2+^, Fe^2+^, and Co^2+^. However, this fluorescence sensor still satisfies Zn^2+^ fluorescence response in a wide range of pH, between pH 4 and 10. 

**Figure 6:**
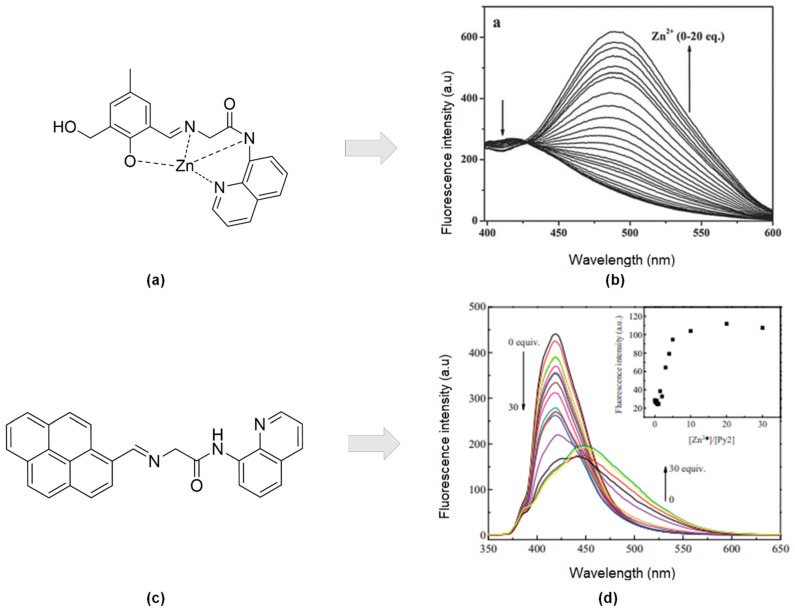
(**a**) Formation of (E)-2-((2-hydroxy-3-(hydroxymethyl)-5-methylbenzylidene)amino) -N-(quinoline -8-yl) acetamide-Zn at ratio 1:1. (**b**) Fluorescence spectra of L1 (10 mM) was titrated with Zn^2+^ (0–20 equiv) in buffer (CH_3_CN–Tris-HCl buffer solution (50 mM Tris, 50:50, *v*/*v*, pH 7.2). (**c**) Structure of (E)-2-((pyren -1-yl methylene)amino)-N-(quinolin-8-yl)acetamide (Py2). (**d**) Fluorescence spectra of 10 μM Py2 in aqueous solution (water-EtOH) in the presence of different concentrations of Zn^2+^ (0–30 equiv), λ_ex_ = 340 nm. Inset: Plots of fluorescence responses changes upon binding to different concentration of Zn^2+^ at λ_em_ = 500 nm.

The development of an aminoquinoline-based fluorophore for zinc sensors has been explored extensively. The combination of carboxamidoquinoline (2-hydroxy-3-hydroxymethyl) -5-methylbenzaldehyde (Figure 6a) was found to improve water solubility. Simultaneously, the sensor displayed remarkable selectivity for Zn^2+^ ions in the existence of other cations in an aqueous solution [4]. A notable 81-nm red-shift of fluorescence emission and enhancement on the fluorescence intensity at 489 nm upon titration between ligands and Zn^2+^ solution (0–20 eq.). A distinct isoemission point at 427 nm was noticeable (Figure 6b). 

High yield of synthesized Py2 by the reaction of pyrene-1-carbaldehyde with quinoline derivative (Figure 6c) was selective to Zn^2+^ ion in ethanol: water solution (95:5 *v*/*v*). Based on the Benesi-Hildebrand equation [91], the binding constant, Ka, of Py2 with Zn^2+^ was calculated to be 5.1 × 10^−4^ M^−1^ with a ratio of one metal-ligand. Moreover, the reversibility of the sensor occurred over EDTA. Both enhancing (500 nm) and quenching (420 nm) fluorescence response of Py2 (10 μM) upon addition of increasing Zn^2+^ (0–30 eq.) in ethanol-water solution (Figure 6d). Regarding interferences studied, no other transition metals interfered at all except for Cu^2+^ [92]. 

**Figure 7:**
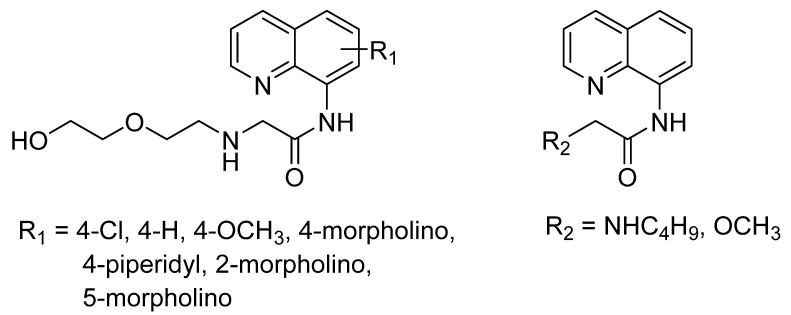
The carboxamidoquinoline-based AQZ family.

A series highly soluble of the carboxamidoquinoline-based fluorophore on the recognition of Zn^2+^ ion in aqueous buffer was investigated (Figure 7) [93]. They reported the affinity’s influences of four different substituents and three various sites on the quinoline ring. As reported by Jiang and co-workers, chelation of Zn^2+^ ion with two N-atoms carboxamidoquinoline produces a five-membered ring due to conjugation increases, and the energy gap between HOMO-LUMO decreases [94]. The coordinated binding still the same as the parent compound similar. However, absorption and fluorescence response varied with a different substituent. These related to the substituent’s behavior, whether it enhanced or prevented the ICT of AQZ upon binding with increasing Zn^2+^. As the position of the same substituent, there is not so much difference in the fluorescence response between 2-position and 4-position. In contrast, 5-position showed no emission with or without Zn^2+^ ion presence due to the carboxamide group [93]. 

The carboxamide group is deprotonated after Zn^2+^ binding, and the 8-aminoquinoline intramolecular hydrogen bond is broken to inhibit the electron-transfer process, which quenches the fluorescence of quinoline [95]. As reported, this carboxamidoquinoline affords two coordination sites for Zn^2+^ ions to bind [96]. Aside from these two sites, introducing a 2-(2-hydroxyethoxy)-ethylamino group recorded an eight-fold rise in quantum fluorescence yield and a red-shift of 75 nm in fluorescence emission when Zn^2+^ was bound in an aqueous buffer solution.

**Figure 8:**
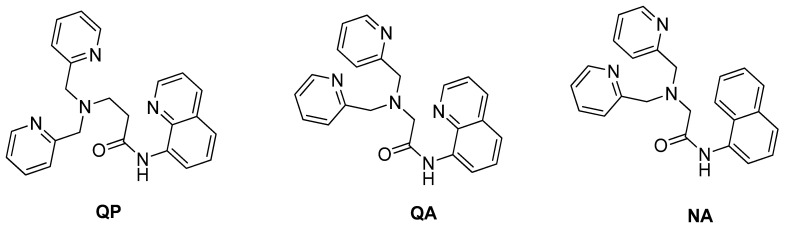
The structures of QP, QA, and NA.

A research study by Lee and co-workers demonstrated the binding complex formation between Zn^2+^ and receptors are versatile. It depended on the receptor itself and the environment of the binding. They proved three different receptors (QP, NA, QA) (Figure 8) gave dissimilar coordination upon binding with Zn^2+^ even though all the receptors were almost similar, which contained 8-carboxamidoquinoline and dipicolylamine (DPA). Generally, Zn^2+^ favored nitrogen of quinoline and nitrogen or oxygen from the amide group and nitrogen from DPA [97]. These results proved to add an amide group into 8-aminoquinoline to improve chemosensor Zn(II). As mentioned before, aside from increasing the water solubility and membrane permeability, the amide group also helps the sensor to be more selective and efficient by growing the binding tendency.

Subsequently, 8-carboxamidoquinoline derivatives started to get attention as fluorophore to detect Zn^2+^ ion, especially in biological fields due to more reports related to carboxamidoquinoline focused on introduction various receptor [98,99,100,101] and the use of it [102,103] and nanoparticle functionalize onto carboxamidoquinoline [104,105,106]. Therefore, a systematic search is needed to better understand the development of 8-carboxamidoquinoline as receptors of the Zn(II) sensor with all due respect to enhanced sensitivity and selectivity for future biological studies application. The interaction and coordination of Zn^2+^ with existing 8-carboxamidoquinoline derivatives differed, requiring many factors to be considered.

## 5. 8-Amidoquinoline Derivatives as Zn Ions Recognition Probes and Their Properties

All the articles reviewed were cross-sectional studies with four sections—first, the function of synthesized, second, titration analysis between probe and zinc ion (fluorescence titration or UV-Vis titration or both), third, the selectivity of the probe sensor with other competitive metal cations, and finally, the limit of detection and reliability of the assessment according to the real sample applications. Most of the papers, starting with the synthesizing 8-amidoquinoline derivatives, then proceed with solution works that are involved titration of the compound with a zinc ion. They continued with certain optimization before undergoes selectivity test with other transition metals such as cadmium (Cd^2+^), copper (Cu^2+^), cobalt (Co^2+^), nickel (Ni^2+^), chromium (Cr^3+^), silver (Ag^2+^), ferum (Fe^2+^), lead (Pb^2+^), and mercury (Hg^2+^). Latterly, they tested the sensor with real samples either from biological or environmental samples that involved requirement detection of zinc as per validation status. However, out of 13 articles, one research does not validate with real sample(s), only three articles tested with water, one article tested with cabbage, and the rest tested with biological samples (Table 2).

### 5.1. Binding Studies of 8-Amidoquinoline Probes via Solution Studies 

Most of the researchers started and trying to develop sensors specifically for the detection of zinc intensively. Among various conventional probes for zinc (II) are derivatives of zinquin [72], Zinbo-5 [73], Zinpyr [74], aminoquinoline [75] and coumarin families [76]. Meanwhile, 8-aminoquinoline and its derivatives [115] were among the first probes developed for zinc (II) detection. They exhibited excellent stability, high affinity to metal ions, and good membrane permeability [41]. However, this zinc (II) chemosensor developed had suffered to differentiate between zinc ion and other competitive cation metals, such as Cd^2+^, Cu^2+^, Co^2+^, Ni^2+^, Hg^2+^, and its 3d^10^ electronic configurations [44,111].

An ideal chemosensor contains a receptor with the most robust affinity binding towards Zn^2+^ ion without interferences (signal-selectivity) of other heavy metals. It has a broad linear responsive range that is highly explored. By inserting a functional group of amide into a conjugated molecule of 8-aminoquinoline, Zn^2+^ ion (borderline acid) is coordinated favorably with aromatic nitrogen atoms (borderline bases), N-amide, and O-amide. Based on this HSAB theory, some researchers [116,117], including us thinking of adding some more nitrogen and oxygen atoms into the 8-aminoquinoline molecule. We believed it could increase the binding affinity towards Zn(II) ion so that it will help to null out the competitive metals that have the same electronic configuration, 3d^10^. 

In determining the binding studies between receptor 8-amidoquinoline compounds, all the papers reviewed were reported on characterization spectroscopy, fluorescence titration, and UV-Vis titration [42,112,118]. They seek binding mode via job plot method and binding constant (K_d_) or association constant (K_a_). However, not all papers were recorded for job plot data. The binding modes were summarized in Table 2. Out of 13 papers, six of them do not conduct the job plot analysis. The left articles stated binding mode between probes and zinc ion was one to one ratio except for research studied by Zhang and co-workers [107], with a ratio of 2:1.

Other than fluorescence and UV-Vis titration, some researchers reported on NMR titration and X-ray crystallography as methods for binding studies between the ligands and Zn^2+^ ion. Both of these studies could help in proposing and determining the binding modes of coordination of metal complexes. As in 8-amidoquinoline derivatives reported, which atom(s) or position(s) had interaction (directly or indirectly) upon binding with Zn^2+^ ion. Therefore, these methods also crucial in metal-ligand binding characterization methods as fluorescence and UV-Vis titration. The combination of fluorescence and UV-Vis titration (gave information on the changes in absorption, emission, and wavelength, provide binding constant and binding ratio) and NMR titration and X-ray crystallography (supported in understanding on how and where they bind) necessitating to get a better experience in determining the interaction between receptors and analytes. 

Molecules containing bonding and non-bonding electrons (n-electrons) can absorb energy in the form of ultraviolet or visible light to excite these electrons to higher anti-bonding molecular orbitals [119]. The more quickly the electrons are excited (i.e., the lower the energy difference between HOMO and LUMO), the longer the light wavelength they can absorb. This is the basic principle of UV-Vis spectroscopy, and it is also applied to the UV-Vis titration method for host-guest complexations [120]. The titration method of UV absorbance has the advantages of quick determination and effortless efficiency. It is likely to become the latest approach to studying interactions between ligands and trace metal ions [121]. Much information could gain from this UV-Vis titration method, such as molar absorptivity or extinction coefficient, changes of absorption (increase or decrease), binding constant, constant rate, job’s plot result (binding ratio), and isobestic’s point in kinetic measurement [122]. 

As fluorescence titration, the purposes are just as same as UV-Vis titration, except it reads the solution’s emission when comparing. Therefore, fluorescence titration could obtain binding interaction of ionophore towards metal [123]. As mentioned by Tamayo and co-workers, spectrophotometric and spectrofluorimetric titration was prepared by the same methods [124]. The stock solutions of ligand and metal were prepared separately by diluting the same concentration. The ligands and metal titration were done by adding the metal ions before proceeded with absorbances and emission reading.

NMR provides specific abilities to characterize ligand molecules that bind enough to be rapidly exchanged between attached and independent state binding modes [125]. Within spectroscopy, NMR spectroscopy plays a vital role in many areas of chemistry and related sciences. The reason for this is that the spectra can be very easily interpreted. In theory, the number of peaks in that spectra could analyze the number of atoms present in the solution. Sometimes, the position of those atoms can be predicted by running a variety of NMR methods. Other than that, NMR also plays a dominant role in studying complex coordination [126]. As we can see, many researchers reported on NMR binding studies of metal-ligands by analyzing the chemical Shift’s change of the presented peaks in ligand upon binding with increasing metal. They proposed that the interaction happened at the peaks that shifted more downfield or upfield once the proportion of the metal increased [42,97,110,111].

**Figure 9:**
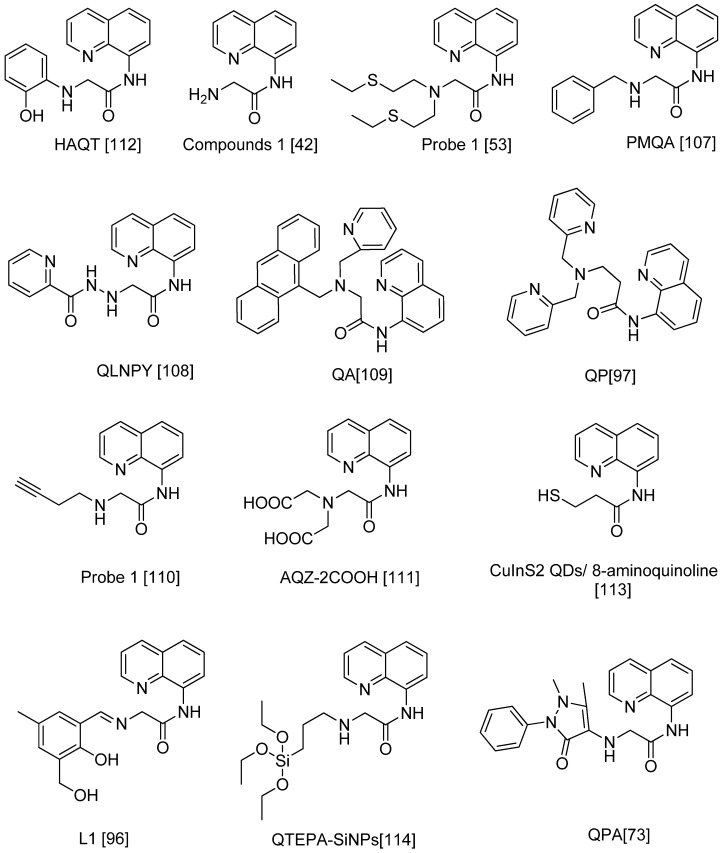
Chemical structures of 8-carboxamidoquinoline derivatives based on Table 2.

As in ^1^H NMR titration of the ligand’s stock solution with various of the equivalent of stock solution of Zn^2+^ ion (0–1.0 eq.), NMR spectra showed NH proton at 11.34 ppm gradually absent upon increase the equivalent of Zn^2+^ ions. Another noticeable change, a new peak of OH, appeared at 10.01 ppm as pile up the volume of Zn^2+^. They suggested that the ligand (Probe 1) probably coordinated with the Zn^2+^ ion in the imidic acid form resulted from tautomerization in acetonitrile. The benefit of Probe 1 (Figure 9) was demonstrated with good selectivity to differentiate between Zn^2+^ and Cd^2+^ through two tautomers, amide and imidic acid [53]. The resonance of amide that was inserted into 8-aminoquinoline offered different binding modes and coordination complex. Therefore, the fluorescence response signal varied according to the introduced metal or Zn^2+^. In Song and co-worker’s case, other cations could have different binding behavior. Aside from that, the solvent also significantly affected the fluorescence response where Zn^2+^ in water, THF, DMSO, ethanol, and Cd^2+^ only in CH_3_CN [110].

Research by Boonkitpatarakul and co-workers also focused on distinguished between Zn^2+^ and Cd^2+^ by using Compound 1 (Figure 9) that contained amidic acid. Deprotonation of amidic proton of Compound 1 upon binding with Zn^2+^ resulted in a large bathochromic shift of UV-Vis absorption from 300 nm to 344 nm. This indicated greater electron delocalization. Zn-Compound 1 binding gave a substantial fluorescent enhancement and shifted from 420 nm and 504 nm. The binding involved strong CHEF, PET of the quinoline’s amino group, and excited-state intramolecular proton transfer (ESIPT) from the amidic proton to N atom of quinoline. The coordination geometry of the Zn^2+^ complex was distorted square pyramidal by three chelating nitrogen from one ligand, one oxygen from another ligand, and one more oxygen of NO^3−^ anion [42]. 

Proton chemical shift changes on methylene of DPA and amide N verified Zn^2+^ ions bound to QP (Figure 9) by coordinating with carbonyl oxygen (C=O). The peak of methylene protons of DPA split into doublets and more deshielded upon addition of Zn^2+^ ion equivalent. These methods were tried in various solvents like methanol, acetonitrile, DMSO, and acetonitrile/water mixture. The result was different since the N-H proton moves towards the downfield in DMSO while vice versa when in acetonitrile. However, once over two equivalent of Zn^2+^ proton of amide (N-H) shifted upfield. Therefore, the binding position suggested taking place was carbonyl O since the proton changes recorded at neighbour proton (methylene DPA and N-H amide) [97].

^1^H NMR titrations in CD_3_CN suggesting three N atoms of amino groups of QLNYP (Figure 9) were coordinate with Zn^2+^ ions since the chemical Shift of secondary amine’s proton were shifted and gradually missing by increasing Zn^2+^ ion equivalent up to 2.0 eq. In the meantime, protons of quinoline, pyridine, and methylene group showed downshift. The peak changes in the position of -CH_2_ deduced the neighbour of the methylene group might be the one that coordinated with Zn^2+^ ions [108]. This suggestion is like a deduction of proton chemical shift changes on DPA from research done by [97].

Given the advantage of fully water-soluble AQZ-2COOH (Figure 9) due to carboxyl groups’ insertion, make it a promising receptor for testing Zn^2+^ in aqueous solution [111]. Crystallography result of binding between Zn^2+^ and AQZ-2COOH confirmed Zn^2+^ formed five-coordinated with three N atoms of pyridine, amide, and amine and another two O atoms of carboxyl groups. These five-membered chelation rings made the Zn-complex more stable. The strong and red-shift of UV-Vis absorption explained the deprotonation of amide moiety, reducing the HOMO-LUMO energy gap and the electron transfer reaction of heterocyclic ligand-metal [111].

Fluorescence enhancement of HAQT (Figure 9) increased proportionally with the increasing zinc concentration up to 10 μM by five-fold at 488 nm, and further addition did not record any further enhancement. Zn^2+^ was proved coordinated with the HAQT via infrared spectroscopy. It noted the disappearance of OH group at 3419 cm^−1^, amide carbonyl absorption slightly shifted to 1656 cm^−1^, and significant difference of N atom of pyridine group at 1300 cm^−1^ and 755 cm^−1^ characteristic hydroxyls [42]. Thus, the plausible reaction between HAQT-Zn involved the deprotonation process that increases electron-donating from N atom of 8-amino to quinoline ring and ICT process that transfers an electron from N atom of the pyridyl group to the metal ion.

Deprotonation and ICT process of L1-Zn (Figure 9) also like the plausible interaction of HAQT-Zn. Fluorescence intensity remarkably shifted from 408 to 489 nm with a quantum yield of 0.138. As in, the isoemissive point was observed at 427 nm. L1 exhibited weak fluorescence, while the coordination of L1 as a ligand to metal gave strong fluorescence [75]. Slightly different from the QPA receptor, other than the transferred electron of N-atom from quinoline, it is also involved in breaking an intramolecular hydrogen bond. The chelation ring formed is more firmed and rigid. Therefore, the fluorescence emission spectrum showed a new emission at 500 nm with a 106-fold rise [73].

Surprisingly, the crystal structure of PMQA-Zn shown uncommon evidence of binding between 8-aminoquinoline and Zn^2+^. Neither N atom of 8-aminoquinoline was bound to zinc even though PMQA (Figure 9) formed a hexadentate chelate ring with Zn^2+^. Those N atoms are critically coordinated to Cu^2+^ perfectly. PMQA-Cu complexes are more stable than PMQA-Zn because ligands containing nitrogen ligands usually have a higher affinity with Zn^2+^, Cd^2+,^ and Cu^2+^. Even though the fluorescence emission quenching when introduced to Cu^2+^, this PMQA also provides information for elucidating zinc probes to prevent Cu^2+^ interferences [107].

A novel and simple turn-on fluorescent were developed by embedded modified 8-amiquinoline (Figure 9) and on the surface of semiconductor nanocrystal quantum dots (QDs), QDs-carboxylic groups. Covalent bonds linked the carboxylic groups of CuInS2 QDs and 8-aminoquinoline at NH_2_ groups to form the CuInS2 QDs/8-aminoquinoline conjugate. Next, upon introducing of Zn^2+^ ions into CuInS2 QDs/8- aminoquinoline conjugate system, the lone pair of electrons of N atoms makes the hole-transfer mechanism inhibit and enhanced the fluorescent of CuInS2 QDs/8- aminoquinoline [113]. Therefore, the fluorescence intensity of CuInS2 QDs/8- aminoquinoline conjugate in the total concentrations of Zn(II) was enhanced with the addition of Zn^2+^ ions.

The ligand added for 8-carboxamidoquinoline consisted of either nitrogen, oxygen, and/or sulfur due to existing of lone pairs of electrons for each atom (Figure 9). These three atoms may react as electron-donating during the coordination binding with Zn^2+^. However, Zn^2+^ does not behave reliably either as a soft or as a hard Lewis acid. It poses a borderline case with no particular fondness for oxygen, nitrogen, or sulfur-donor ligands to be coordinated. The results of the concluded binding coordination sites to form complexes were different. Roughly, we reviewed all of them as there were no fixed preferences and they differ based on derivative, atoms presenting, molecular geometry, medium, and environment. Therefore, the results might contradict. Nevertheless, the importance of development in sensing Zn^2+^ was successful since the different enhancement for fluorescence upon binding with Zn^2+^ was recognized.

### 5.2. Detection Limits for Zn^2+^ Ions and Possible Interferences

In analytical chemistry, the detection limit or, in other terms, is the limit of detection (LOD), defined as the lowest concentration of target substance reliably distinguished from the blank [127,128,129]. As we mentioned earlier, the purpose of this systematic review was to summarize the research design and outcomes of the recognition of zinc ions by 8-amidoquinoline. Herein, the lowest concentration of analyte zinc ion detected was reported. Based on our review, the LOD range of the included articles was between 3.36 × 10^−8^ M to 445 × 10^−8^ M. Therefore, the detection limit of 8-amidoquinoline-based zinc chemosensor was in nanomolar proven these derivatives are highly sensitive to recognized Zn(II) ion.

There are potential interferences of transition metals, especially metals that belong to the same group (Group 12) or same period (Period 4) as Zn(II) ion in the periodic table. They showed almost similar characteristics to Zn(II), 3d^10^ configuration. Therefore, Zn(II) detection had suffered from the cross-interference of Cd^2+^, Cu^2+^, Co^2+^, Ni^2+^, and Hg^2+^ [130,131,132]. Based on [71], when the chelation of a metal-ligand complex coordinated from a five-membered to a six-membered ring, ligands usually selective for the smaller metal ions compared to the larger metal ions. For example, TQA ligands are more selective to metal Zn^2+^ (smaller radius) than Cd^2+^ (larger radius). In this research, they demonstrated the selectivity of the ligand to the metal ion radius. They concluded, good metal-ligand binding depends on two factors that are the size of metals ion and the size of chelate rings. 

Thus, all the included articles performed a screening and analysis of 8-amidoquinoline derivatives on the study of the interference of the other heavy metals towards zinc detection—evaluations between those studies on the interference of the transition metals are documented in Table 2. All of the studies recorded interference. Trace metals like Cd^2+^, Cu^2+^, Co^2+^, Ni^2+^, Hg^2+^, Fe^2+^, Pb^2+^, and Cr^3+^ interfered in the detection of zinc using 8-amidoquinoline derivatives. The only difference in those studies is the interference metals and the metal ratio to zinc used. Nevertheless, certain studies [53,72,96,109,110,112,113,133] demonstrated that the sensing probe could be used in a real sample due to the metals involved not present or least in biological applications. These exhibited 8-amidoquinoline derivatives are selective to zinc detection due to minor interferences issues. 

### 5.3. The Reliability of Zn Fluorophores for Zn Ion Determinations

As per the development of any analytical methods like fluorescence sensor, it will require some validation assessments and testing with real samples to ensure consistency, accuracy, and applicability. By definition, validation is to verify that the analytical method is appropriate for its intended purpose. The analyte can be tested correctly, clearly, and precisely over the specified range [134]. After the sensor’s optimization, performances of the sensors that comprise among these parameters (calibration curve, linear range, detection limits, selectivity, specificity, repeatability, reproducibility, reversibility, stability, analysis time, response time, and others) needed to be done as validation [135]. As for the chosen papers, validation for specific parameters was done after each optimization step. They also test the fabricated sensor with a real sample that has been selected, as mentioned before, in Table 2. As the detection of Zn^2+^ ions is crucial for biological purposes, eight of 13 articles were tested with biological samples involving cells. The remaining papers were tested with river or tap water and cabbage. 

### 5.4. Possible Mechanisms and Interactions Involved 8-Amidoquinoline Derivatives upon Binding with Zn^2+^

**Figure 10:**
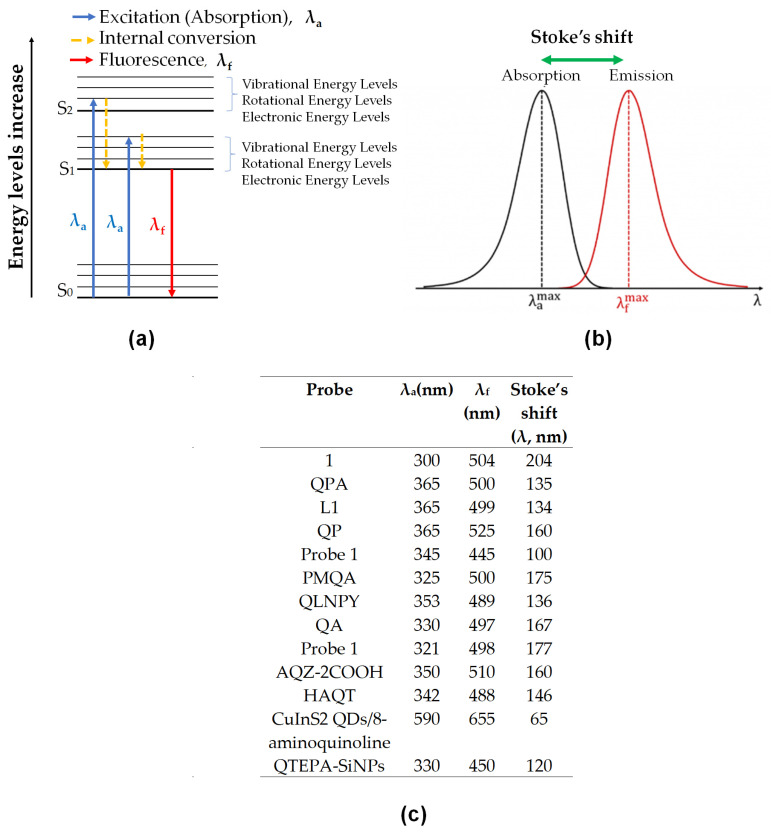
(**a**) Simplified Jablonski diagram energy level; where the blue arrow is excitation wavelength maximum and the red arrow is emission wavelength maximum. (**b**) The diagram on how to identify Stoke’s Shift, in wavelength (nm). (**c**) Table of details absorbance, emission and stoke’s Shift for all probe in Table 2; Stoke’s Shift calculated from the difference between emission and absorbance wavelength.

Fluorescence occurs when a substance can absorb energy from photons and emit the energy as fluorescence by internal conversion mechanisms and losing the energy from an excited state to ground state level. It is indicated for better visualization and explanation on a simplified Jablonski diagram (Figure 10a). The diagram showed the increasing energy level from the ground state (S_0_) to the excited state (S_1_) and an excited singlet state (S_2_). The excitation from S_0_ to S_1_ or S_2_ depends on the photons’ energy given during the excitation and absorption process. The energy absorbed will then undergo vibrational relaxation by dissipating energy into the surrounding medium before released to S_0_. The losing energy involved the transition of higher to lower electrical energy and lower to higher vibrational energy. These changes made the substance fluoresce [136,137]. From Jabslonki’s diagram, we can say that the longer the wavelength, the lower the energy, and vice versa.

Stokes shift is a difference between absorbance maximum and emission maximum. The calculation of this may present in wavelength or wavenumber (Figure 10b). In some instances, it is essential in determining the value of the Stokes shift, especially in the biological application. The smaller distance between excitation and emission wavelength can hardly distinguish the wavelength, and the spectral will significantly overlap. As we can see in Figure 10c, the table showed the difference between the two wavelengths were in the range of 65–204. It is considered wide Stokes shift for all the probes, and therefore, they were suitable in testing the bio-real samples.

**Figure 11:**
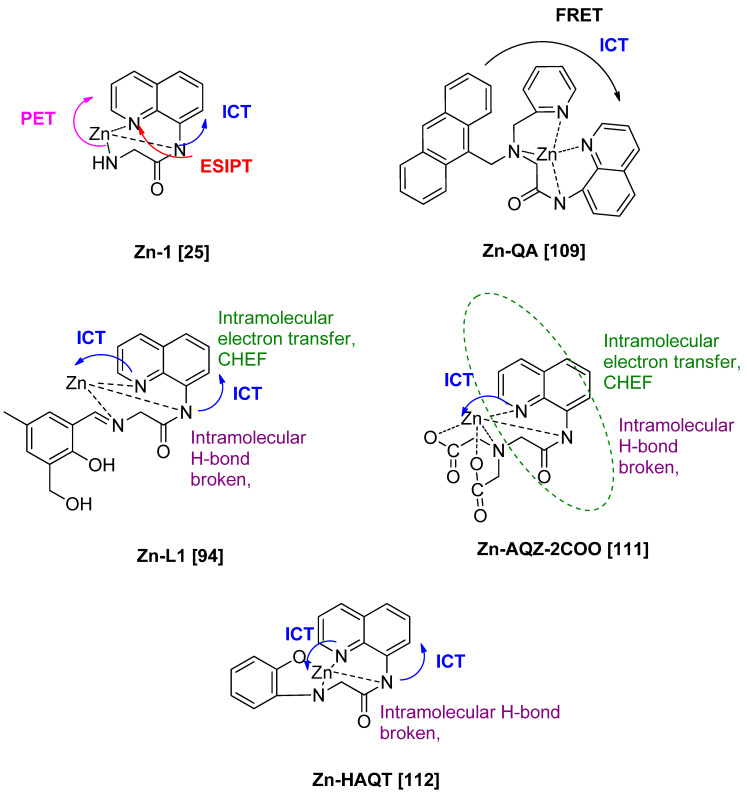
Possible mechanisms and interactions involved 8-amidoquinoline derivatives upon binding with Zn^2+^.

Those are the basic principle of interactions in fluorescence spectroscopy. Next, the photophysical mechanisms for the fluorescent probe of 8-amidoquinoline for Zn^2+^ ions involve CHEF, ICT, PET, ESIPT, and FRET signals. Among the chosen articles, some probes used multi-signal mechanisms, and others using conventional mechanisms (only one). It should be noted that a combination of two or more mechanisms sensors produces much better results. Figure 10c shows that the largest Stokess shift was Compound 1 by Boonkitpatarakul and co-workers [42]. The more significant difference may be due to the (1) strong CHEF signal upon binding with Zn^2+^, (2) PET process of N-atoms from amino group to the fluorophore, (3) ESIPT mechanism of H-amidic to N-quinoline, and ICT of the complex Zn-1 (Figure 11).

As for QA-Zn, we believed the FRET mechanism occurred. It was between fluorophore anthracene and fluorophore quinoline due to the spectral of anthracene (emission) was overlapped with quinoline (excitation) [109]. In this case, anthracene became a donor while quinoline became an acceptor. Figure 11, probes L1, AQZ-2COOH, and HAQT showed dual signal mechanisms, CHEF and ICT [96,111,112]. Upon binding with Zn^2+^, intramolecular hydrogen bonding of 8-amino broken. It leads to the intramolecular transfer of electrons of N-atom of the amide to quinoline (CHEF). Other than that, ICT mechanisms were involved between N-quinoline to Zn. These will enhance the intensity of the fluorescence. The mechanisms involved between 8-amidoquinoline derivatives upon binding with Zn almost have the same signals and more than one signal.

### 5.5. Another Recent Studies Related to 8-Amidoquinoline Derivatives as Fluorophores for Zinc’s Detection

Zn^2+^ sensors based on fundamental carboxamidoquinoline have rapidly developed because of simple synthesis, binding ratio, and ratio detection. Zebin and co-workers linked functional magnetic core-shell fibrous silica material (AQ-Fe_3_O_4_@SiO_2_@KCC-1) and amidoquinoline group with piperazine to get high selectivity and enhanced the fluorescence intensity in aqueous solution [138]. As for combination of diarylethene based sensors with carboxamidoquinoline unit were reported by three groups, (1) Diarylethene-based sensor with a carboxamidoquinoline unit (linker-piperazine) that can differentiate Zn^2+^ and Cu^2+^ [46], (2) Consist of diarylethene with a benzyl-linked 8-aminoquinoline-2 -aminomethyl pyridine reported naked eye Zn fluorescent chemosensor [139], (3) Ratiometric and reversible of fluorescence Zn sensor based on diarylethene and carboxamidoquinoline unit [140].

Hydrogen bonds are readily formed by the nitrogen center in quinolines, rendering those derivatives weak in fluorescent intensity in protic polar solvents. However, as mentioned above, the coordination between NH-amide and Zn would induce ICT and ESIPT mechanisms and inhibit the PET process resulting in enhanced fluorescence intensity (CHEF). Other than that, a metal complex coordination in the presence of an amide-containing ligand along with a carboxylate ligand will boost the self-assembly required for an emission induced by aggregation. This mechanism is also known as aggregation-induced emission (AIE) [141].

**Figure 12:**
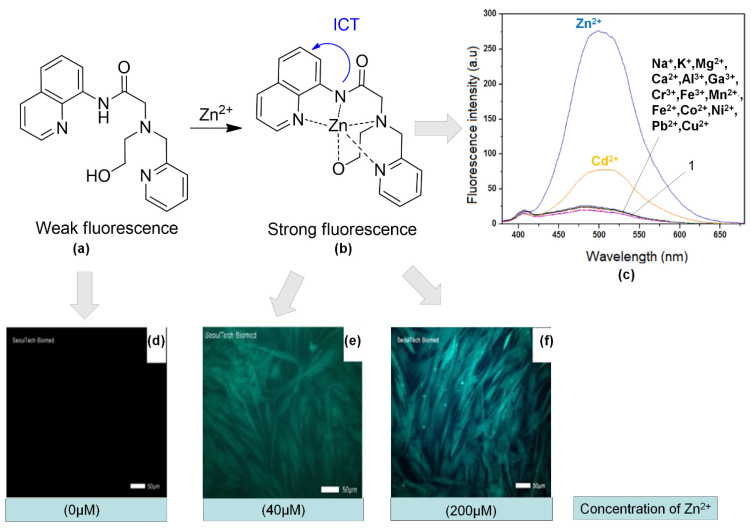
(**a**) Structure of 1. (**b**) Fluorescence enhancement mechanism and proposed structure of 1 upon binding with Zn^2+^. (**c**) Fluorescence spectra of 1 in the presence of different cations in 10 mM bis–tris, pH 7.0 buffer (λ_exc_ = 356 nm). Fluorescence images of fibroblasts cell that exposed to Zn^2+^ at different concentrations 0 (**d**), 40 (**e**), and 200 (**f**) μM for 4 h and then cultured with 1 for 30 min.

Simple chemosensor 1 (2-(N-(2-hydroxyethyl)-N-((pyridin-2-yl) methyl)amino)-N-(quinoline-8 -yl) acetamide) (Figure 12a) consists of both the binding site and the water-soluble functional group of quinoline as fluorophore and 2-((pyridin-2-yl)methylamino)ethanol. The probe showed changes from weak fluorescence to strong fluorescence upon binding with Zn^2+^ (Figure 12a,b). In Figure 12c, we can see that other than Zn^2+^, Cd^2+^ also showed the enhancement of fluorescence intensity at wavelength 500 nm. However, both intensities could be compared. Therefore, this chemosensor demonstrated its potential in monitoring Zn^2+^ in human dermal fibroblasts. We could see that the fluorescence imaging of the cells with 1 at different concentrations of Zn^2+^ was significantly enhanced (Figure 12d–f) [142].

**Figure 13:**
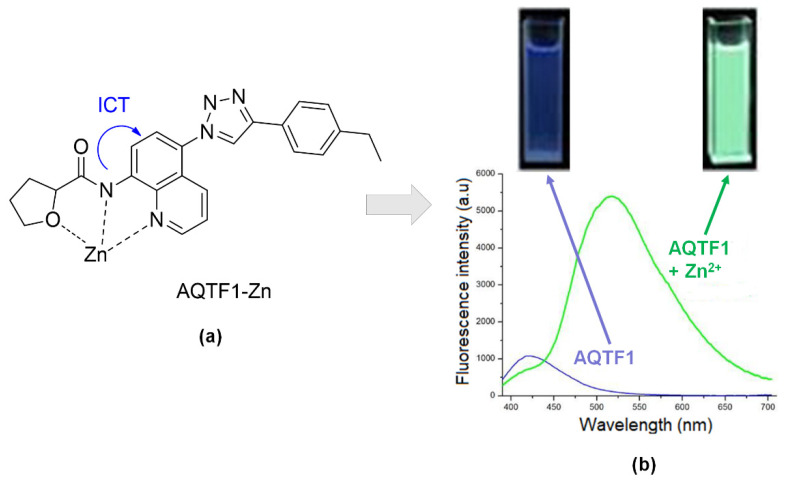
(**a**) Complexation of AQTF1-Zn. (**b**) Naked eye color changes under visible fluorescence and their fluorescence intensity spectra, blue (without binding with Zn^2+^) and green (upon binding with Zn^2+^).

A novel fluorescent sensor of AQTF1-Zn (Figure 13a) made up of N-(quinolin-8-yl) tetrahydrofuran-2- carboxamide moiety demonstrated color changes under a UV lamp long-wavelength. The color was enhanced upon adding the Zn. Therefore, the fluorescence intensity of AQTF1 lower compared to AQTF1-Zn (Figure 13b). The probe was tested at fluorescence emission at 511nm in solvent DMSO-water at pH 7.4, 1:1. This condition makes this sensor suitable for the Zn testing cell imaging in vivo. Du and co-workers also stated this sensor produced high selectivity to Zn^2+^ ions without interference from Cd^2+^ ions. [143].

**Figure 14:**
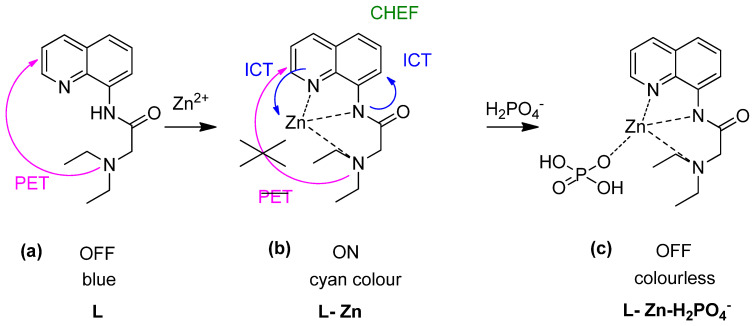
“Off–On–Off” fluorescent chemosensor for (**a**) L, (**b**) L-Zn, and (**c**) L-Zn- H_2_PO_4_^−^ for relay recognition in an aqueous environment.

Followed by AQTF1-Zn as a type of fluorescent chemosensor for the relay recognition of Zn^2+^ and H_2_PO_4_^−^, Kumar and co-workers explored “Off-On-Off” fluorescent chemosensor based on the receptor 2-(diethylamino)-N-(quinolin-8-yl) acetamide (L) for the same relay’s detection (Figure 14a) [144]. Zn^2+^ ions and phosphate are two necessary biomolecular involved in DNA and RNA, pharmaceutical drugs, and therapeutic [145]. Other than that, excessive H_2_PO_4_^−^ in the environment will cause algal growth that will lead to decomposition and reduce the dissolved oxygen levels and lower the water quality [146]. Upon interaction with Zn^2+^, three N-atom formed complexation with Zn^2+^, led to PET’s inhibition, and promoted ICT and CHEF. Therefore, the color intensity was changed from blue to cyan under visible fluorescence change by the naked eye (Figure 14a,b). The metal complex may then turn off fluorescent upon binding with H_2_PO_4_^−^ (visible under UV light–colourless) (Figure 14c).

**Figure 15:**
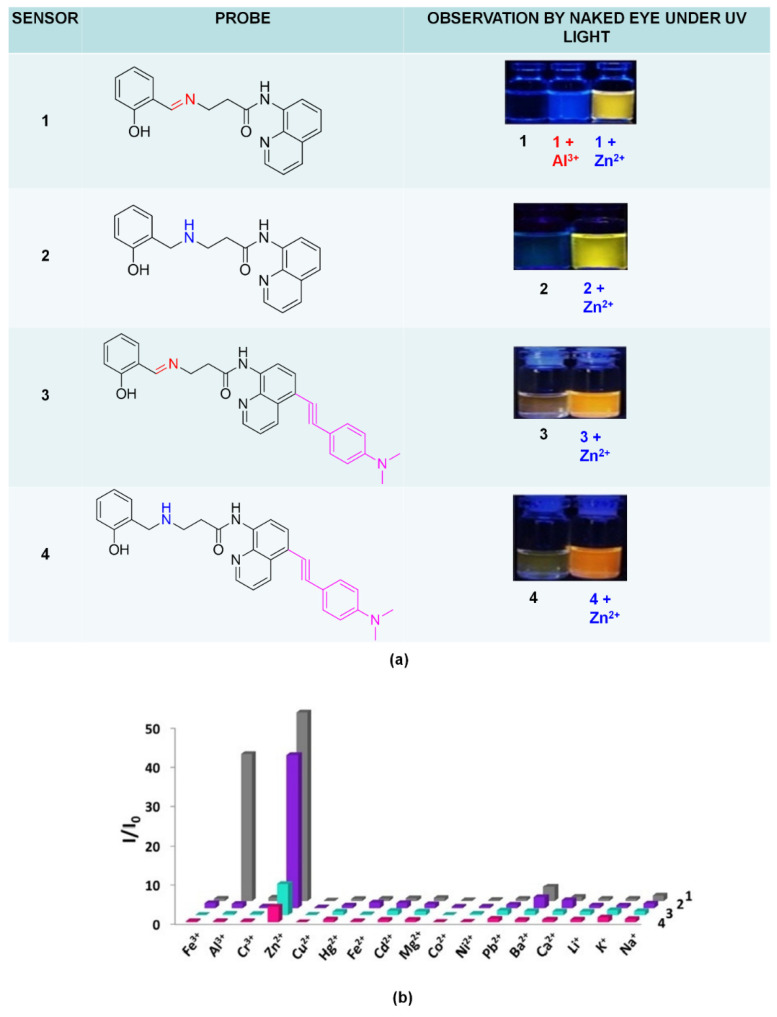
(**a**) Structure of four sensors and observation of color changes before and after reaction with metal ions. (**b**) Fluorescence enhancement ratios of sensors 1 to 4 in 9 EtOH: 1 H_2_O in the presence of different metal ions at excitation 360 nm and emission 420 nm (200 mM).

Vongnam and co-workers proved that emissions’ impacts depended on the structure; even the structure consisting of 8-amidoquinoline and salicylaldimine moieties, slightly different in structure, will produce different results (Figure 15a). These salicylaldimine group 1) lowered the fluorescence intensity by isomerization of C=N and PET, 2) Formed pentacoordinate complex with Zn^2+^ via chelation. The range of detection limits for the four sensors was recorded from 0.024 to 0.431 mM, and among the four sensors, only sensor 1 could be fabricated as a paper-strip fluorescent chemosensor. Sensor 1 showed changes in intensity upon binding with Al^3+^ ions (Figure 15a). However, the emission was at different wavelength (Al^3+^ = 439 nm, Zn^2+^ = 550 nm). Figure 15b) displayed fluorescence enhancement ratios of sensors 1–4 in 9 EtOH: 1 H_2_O in the presence of different metal ions, respectively [147].

### 5.6. Limitations of and the Future of Quinoline and Derivatives Fluorophore for Zn Ion Analysis 

This review has made an effort to systematically analyze the existing literature on the 8-amidoquinoline derivatives as fluorophore in detecting Zn^2+^ ions (2010–Jan 2020). As a result, 13 articles were analyzed and discussed in this review. The developing and synthesizing of highly sensitive fluorescent probes for zinc recognition, especially in the biological field, is expected to be ongoing. Based on the recent exploration on the 8-amidoquinoline to improve cell permeability and HSAB theory’s rule, we believed N-atom and O-atom in 8-amidoquinoline derivatives could be an excellent probe in detecting Zn. The identification of 8-amidoquinoline derivatives conducted for decades may help to elucidate and plan future studies. According to HSAB theory’s rule, as borderline acid, zinc will be preferred to bind to borderline bases like aniline, pyridine, N^3−^, Br^−^, NO^2−^, SO_3_^2−^, N_2_. As in Figure 9, at least one of these borderline bases is added to the 8-amidoquinoline combination structure.

We summarized that fluorescent chemosensors of Zn^2+^ based on 8-amidoquinoline succeed in improved water solubility and membrane permeability as most of the applications were tested with biological samples. Nevertheless, some of these sensors showed that Zn^2+^ could be distinguished with Cd^2+^, the most competitive metal ions, since it is in the same group as Zn^2+^. Development of these sensors also provides visibility by naked-eye on color changes before and after binding with Zn^2+^ under long-wavelength (365 nm). The apparent color changes would help in determining the presence of Zn^2+^ ions without using the instrument. It also could be used as preliminary results before proceeding to the next steps.

However, some limitations still exist for these compounds as Zn fluorophores. All of the fluorophores suffered some interferences, especially in higher concentrations of the interfering ions. Thus, some of the binding mechanisms were not as expected, probably attributed to the ligand and steric hindrance position in forming five chelation with zinc. Some studies showed the most affected on the fluorophore changes were environments like the type of solvent and environment of the target ions. These fluorescent probes may be explored more by changing the chemical structure. We can also confirm from this analysis that it does not mean that a probe with a rich borderline base will offer the best zinc detection performance.

## 6. Conclusions

Fluorescence is best known for its high sensitivity, repeatability, low detection limits, simplicity, and potential to allow real-time monitoring due to their quick response times. These characteristics made fluorescence sensing of metal ions one of the most potent detection tools to be explored. In this review, we covered tremendous interest in 8-amidoquinoline as Zn^2+^ probes in fluorescent sensor design reported in the last few years, and the output of their research was discussed. Zinc sensors were involved via various binding modes and different fluorescence response mechanisms based on the system and experimental conditions such as the type of metal salt used, type of pH, type of buffer, and solvent used, and the analyte’s concentrations. However, all the selected sensors were stilled, allowing the selective detection of Zn^2+^ ions in various environmental or/and biological samples. Most of the receptors use nitrogen as the binding site as it is favorable and form relatively simple geometries with Zn^2+^. Based on this review, there is still much to improve regarding the derivative of 8-amidoquinoline as a receptor, such as fast response and bio-compatibility, especially for biological applications. Further studies are suggested for a better understanding of the diversity of the coordination complexes and geometries. This review could help to elucidate, design, and explore new 8-amidoquinoline derivatives for future chemosensor applications that are more selective and sensitive to zinc ions.

## Figures and Tables

**Table 2:** The comparisons and assessments of collected studies that related to 8-amidoquinoline derivatives as fluorophore of zinc chemosensor.

Articles	Year	University/Countries	Probe	Binding Mode (Probe: Zn)	Interferences	Detection Limits	Real Sample
[42]	2018	Thailand	1	1:1	Ni^2+^, Cu^2+^, Co^2+^	160 × 10^−8^ M	Cabbage
[73]	2012	The Chinese University of Hong Kong, China	QPA	1:1	Cd^2+^, Fe^2+^, Cu^2+^	13 × 10^−8^ M	HK-1 cells
[96]	2012	Lanzhou University, China	L1	-	Cd^2+^, Ni^2+^, Cu^2+^, Hg^2+^	100 × 10^−8^ M	Human bladder cancer
[97]	2013	Korea	QP	-	Cu^2+^, Fe^2+^, and Co^2+^	-	-
[53]	2012	Lanzhou University, China	Probe 1	1:1	Hg^2+^, Cu^2+^, Cr^3+^, Ag^2+^	8.14 × 10^−8^ M	HELA cells
[107]	2013	Lanzhou University, China	PMQA	2:1	Cd^2+^, Co^2+^, Cu^2+^	8.85 × 10^−8^ M	HELA cells
[108]	2019	Shenzhen University, China	QLNPY	-	Cu^2+^	3.8 × 10^−8^ M	HepG2 cells
[109]	2014	Nanjing Normal University, China	QA	1: 1	Hg^2+^, Cu^2+^	3.36 × 10^−8^ M	Intracellular cells
[110]	2019	Xi’an Jiaotong University, China	Probe 1	1:1	Cr^3+^	6.3 × 10^−8^ M	Tapwater
[111]	2018	Zhoukou University, China	AQZ-2COOH	-	Cu^2+^	10.2 × 10^−8^ M	HeLa cells
[112]	2013	Xinxiang University, China	HAQT	1: 1	Ni^2+^, Cu^2+^	25.6 × 10^−8^ M	River water
[113]	2014	Jilin University, China	CuInS2 QDs/8-aminoquinoline	1:1	Pb^2+^, Hg^2+^	445 × 10^−8^ M	Tap water
[114]	2011	United States	QTEPA-SiNPs	-	Fe^2+^, Cu^2+^,	10 × 10^−8^ M	Yeast cells

## Data Availability

No new data were created or analyzed in this study. Data saharing is not applicable to this article.
